# Supplementary material for: Degassing-induced fractionation of multiple sulphur isotopes unveils post-Archaean recycled oceanic crust signal in hotspot lava
Source: Nat Commun. 2018 Nov 30;9:5093. doi: 10.1038/s41467-018-07527-w (PMC6269480; doi:10.1038/s41467-018-07527-w)
Supplement: Supplementary file 1 — Supplementary Information [file 41467_2018_7527_MOESM1_ESM.pdf]

Supplementary Information for

Degassing-induced fractionation of multiple sulphur isotopes unveils post-Archaean recycled oceanic crust signal in hotspot lava

Beaudry et al.

Supplementary Discussion

Potential matrix effects

The most negative $\delta^{34}S$ values (< -4 ‰) measured in our sulphide inclusions are all from multi-phase (group 2) inclusions with mss–iss–oxide intergrowths (Supplementary Figs. 3b, 4) or S-depleted droplets (group 3) displaying quench textures and producing low total major element contents on electron microprobe analyses (Supplementary Fig. 3c-e). The stoichiometry of the Cu-bearing phase in group 2 sulphides, cubanite ($CuFe_2S_3$), is intermediate between pyrrhotite ($Fe_{1-x}S$) and chalcopyrite ($CuFeS_2$). Cabral et al. (ref. 1) found an instrumental bias on $\delta^{34}S$ for chalcopyrite relative to pyrrhotite of +0.8 ± 0.9 ‰, i.e. small and positive, hence it should not drive measured $\delta^{34}S$ values towards more negative values, assuming that the effect is similar for $CuFe_2S_3$. Natural isotopic fractionation between these sulphides should also be negligible around 1150°C (ref. 2). Thomassot et al. (ref. 3) found important instrumental mass fractionation (IMF) for pyrrhotite and chalcopyrite standards, with α_i values between 0.98784 and 0.99125 in different analytical sessions, but the IMF for chalcopyrite and pyrrhotite was also similar within analytical sessions. The IMF calculated on our repeat analyses of Anderson pyrrhotite (Supplementary Table 1) was significantly smaller, with α_i = 0.99764 for $^{34}S/^{32}S$ ratios, i.e. a $\delta^{34}S$ difference of -2.4 ‰ relative to the expected value, compared to -8.8 ‰ to -12.2 ‰ for Thomassot et al. (ref. 3). Thus, we believe potential matrix effects between pyrrhotite and cubanite to have been negligible and our data to reflect the true $\delta^{34}S$ values of the sulphides. Furthermore, the cubanite-bearing sulphides that we analysed (group 2) show isotopic overlap with the homogeneous mss sulphides (group 1), and did not yield the most negative $\delta^{34}S$ values in our dataset. The group 3 quenched sulphides (n = 4) produced totals of ~95 % on EPMA analyses with lower S content, but are otherwise similar to pyrrhotite in composition (see Supplementary Data 2 and overlap with group 1 in Fig. 4a of main text), and reach the lowest $\delta^{34}S$ value (-9.6 ± 0.4 ‰). If matrix effects for cubanite were significant, one might expect the group 2 and group 3 sulphides, which have important compositional differences, to also have different isotopic fractionation factors. On the contrary, they show similarly large $\delta^{34}S$ ranges (see Figs. 3–5 of main text). In contrast to the group 1 homogeneous mss sulphides, several of the group 2 and 3 sulphides are in direct contact with matrix glass (representative of bulk melt at quench) (e.g. Supplementary Fig. 3d,f), consistent with their re-equilibration with later, more $\delta^{34}S$-negative, degassed melt.

Potential matrix effects due to sulphur speciation differences in melt inclusions and matrix glasses are also likely to be negligible. As noted by Fiege et al. (ref. 4) the energy of sputtered secondary ions largely exceeds that of bond energies in the silicate glass, hence no detectable instrumental fractionation should occur between S^{2-}- and S^{6+}-bearing glasses.

Models considered for sulphur isotope fractionation

Isotopic fractionation models depend on the fractionation factors between sulphur species, which can be determined experimentally. Most models (e.g. refs. 5,6) have used the $\alpha(H_2S_{gas}–S^{2-}_{melt})$, $\alpha(SO_{2\,gas}–SO_4{}^{2-}_{melt})$ and $\alpha(H_2S_{gas}–SO_4{}^{2-}_{melt})$ fractionation factors from the experiments of Miyoshi et al. (ref. 7). More recently, Fiege et al. (refs. 4,8) noted that the $\alpha(SO_{2\,gas}–SO_4{}^{2-}_{melt})$

and $\alpha(H_2S_{gas}–SO_4{}^{2-}{}_{melt})$ values of Miyoshi et al., which were measured in molten salts, may not be suitable for silicate melts. Fiege et al. thus conducted experiments with silicate melts and observed greater fluid–melt S-isotope fractionation, especially at reduced conditions (i.e. around ΔFMQ = 0), where the melt becomes highly depleted in ^{34}S. Figure 6 of the main text shows that the fractionation factors determined by Fiege et al. (ref. 8) approximately double the magnitude of isotopic fractionation around ΔFMQ = 0, relative to those reported by Miyoshi et al. (ref. 7). The isotopic fractionation factor between sulphide liquid and H_2S $\alpha(FeS_{liq}–H_2S_{gas})$ has been taken from ref. 9.

Clearly, while degassing of the El Hierro magma probably started at high pressure and oxidized conditions, fO_2 must have decreased during degassing to cause isotopic fractionation towards negative values. At high fO_2, S in the melt is dominantly dissolved as $SO_4{}^{2-}$, hence degassing preferentially results in the loss of lighter S (H_2S and SO_2). At low fO_2, where most S in the melt is S^{2-}, degassing favours the loss of heavier S, i.e. SO_2. The effect of pressure is significant because larger proportions of H_2S enter the gas at high pressure, lowering the magnitude of S-isotope fractionation since the fractionation between $S^{2-}{}_{melt}$ and H_2S_{gas} is minimal. The model of Fiege et al. produces stronger S-isotope fractionation, consistent with our most negative matrix glass δ^{34}S values, but overestimates the extent of fractionation recorded by S-rich melt inclusions. We used the relationships shown in Figures 2b (fO_2) and Supplementary Fig. 5 (P) to estimate fO_2 (in ΔFMQ units) and P for a given S content, and used those to obtain an evolving $\alpha_{gas–melt}$ for decreasing S content (or Fraction of S remaining in the melt). The SO_2/H_2S ratio in the gas phase was estimated using equation 5 of Marini et al. (ref. 5), and absolute log fO_2 vs ΔFMQ from ref. 10. This allowed us to calculate a range of possible δ^{34}S values following S isotope fractionation during degassing for each model, as shown in Figure 2a and Supplementary Data 3. The equations describing this isotopic fractionation are given by Marini et al. (refs 5,11). It should be noted that water fugacity (fH_2O), rather than pressure, is used in these equations, but both parameters can be used interchangeably: while fH_2O differs significantly from P at pressures >100 MPa, they converge for P <60 MPa (Supplementary Fig. 6), where the pressure effect is most pronounced. We ran our model with both, and there is no noticeable effect. The fractionation trends shown in Figure 2a are calculated with fH_2O values estimated from D-Compress run 1 (Supplementary Fig. 6).

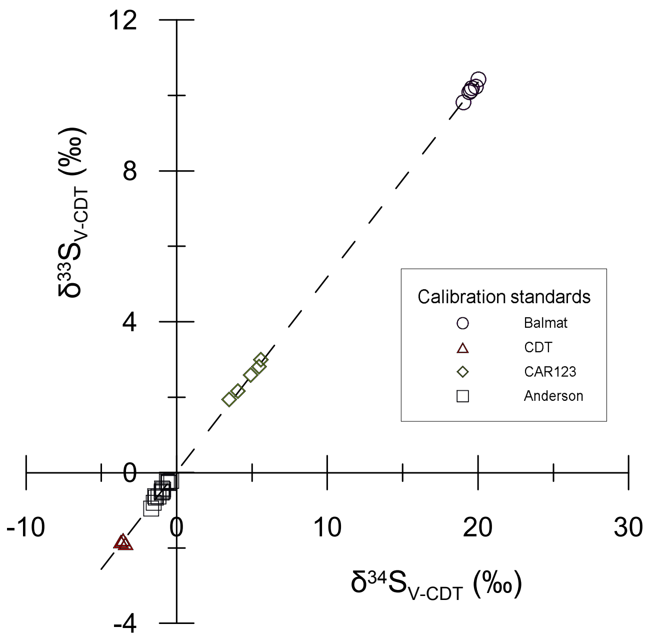

Supplementary Figure 1: Calibration of the $\delta^{33}S$ vs $\delta^{34}S$ regression line with natural sulphide standards. The calibration line has a slope of 0.5166 ± 0.0020 (2σ) in $\delta^{33}S_{V\text{-}CDT}$ - $\delta^{34}S_{V\text{-}CDT}$ space, closely corresponding to the theoretical mass-dependent fractionation slope of 0.515 (ref. [12]). Balmat and CAR123: pyrite; CDT: Canyon Diablo Troilite; Anderson: pyrrhotite. Measured $\delta^{33}S$ and $\delta^{34}S$ values (shown here) are used for the calculation of the slope *k* in equation 5.

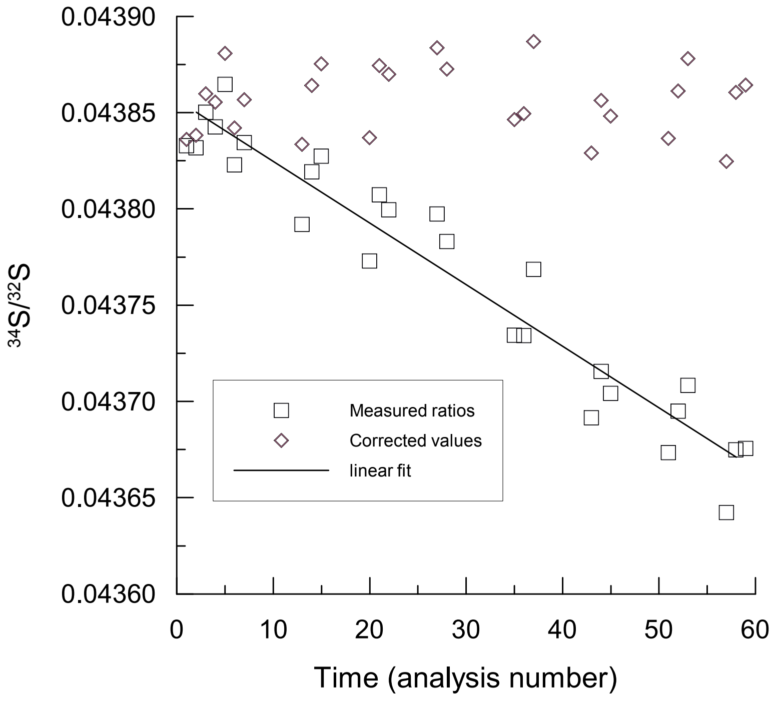

Supplementary Figure 2 Measured $^{34}S/^{32}S$ ratios in our glass standard (P1326-2 basaltic glass; squares) showed instrumental drift on the first day. A linear correction was applied (diamonds) by subtracting the magnitude of the drift, determined by the slope of the linear regression, from the measured isotope ratios. The equation of the regression line is $y = -0.0000032x + 0.043857$, with $R^2 = 0.93$.

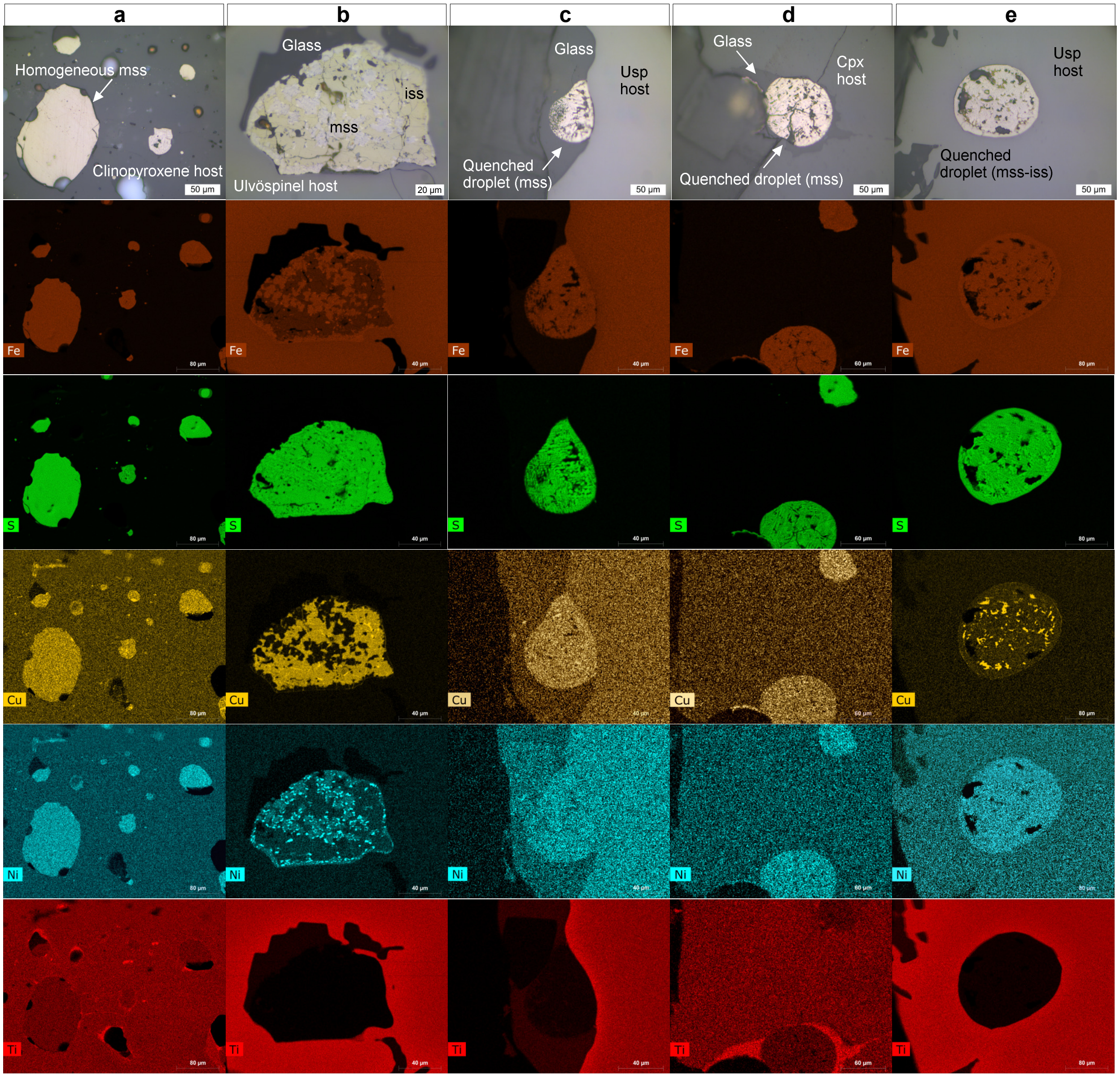

Supplementary Figure 3: X-ray elemental intensity maps for various sulphides. **a,** Group 1 sulphide (EH_4-03-1, see Supplementary Data 2), showing homogeneous surface and compositional maps for all elements shown. **b,** Group 2 sulphide (EH_3-12), showing intergrown mss and Cu-rich iss, with small blebs of a Ni-rich phase within the mss. **c–e,** Group 3 sulphides (c: EH_3-45; d: EH_4-05-3; e: EH_3-28), showing rounded blebs and quench trellis textures (see S maps). One of these (**e**) has an interstitial Cu-rich phase, not probed but assumed to be iss as for group 2 sulphides. It also has a slightly higher Ni and Cu contents (Supp. Table 2, Supp. Fig. 7). Note the Ti zoning of spinel in (**c**), which would be expected if the melt was becoming more reducing in the last stages of crystallization, enriching the boundary in Ti.

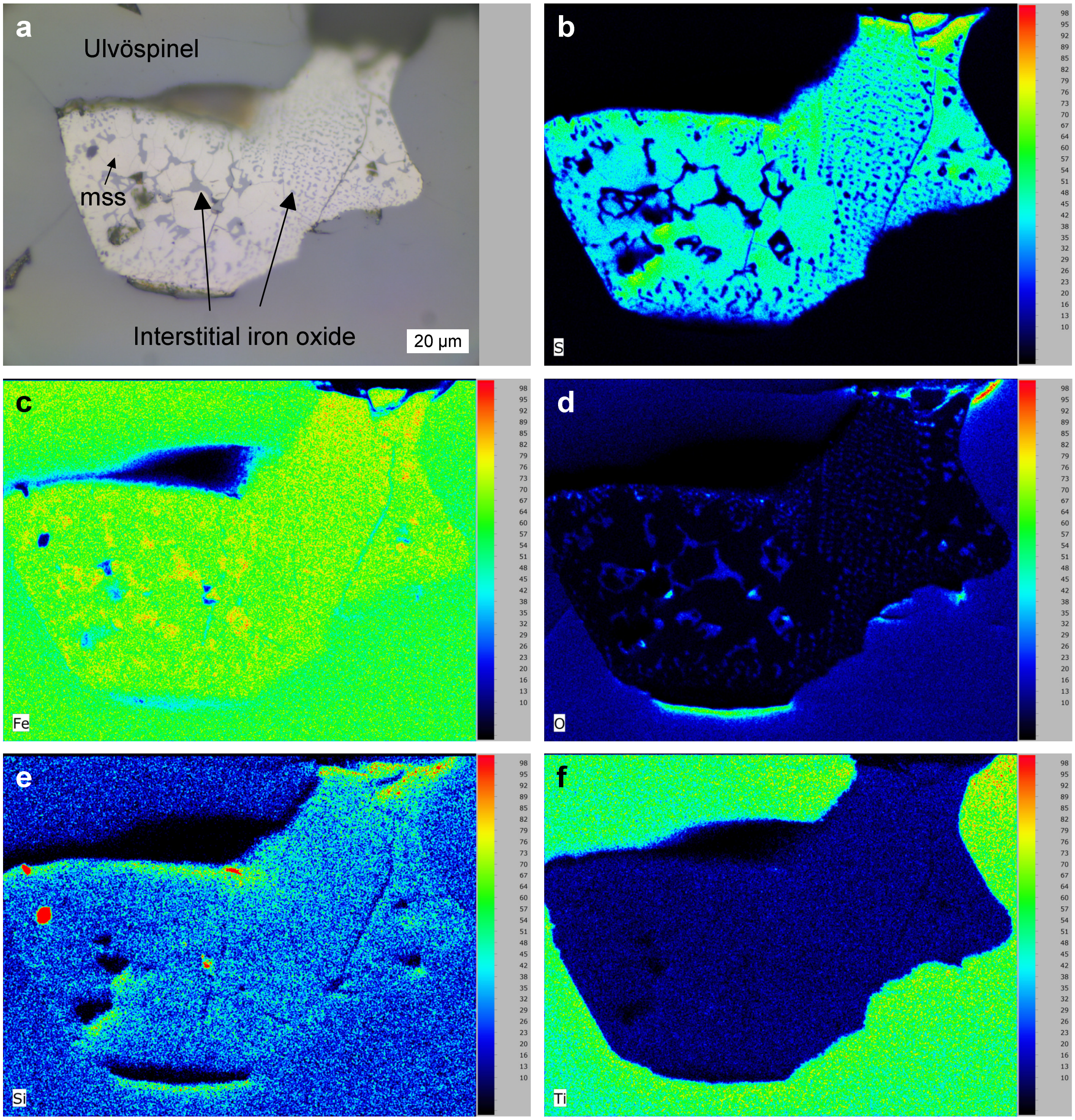

Supplementary Figure 4: X-ray elemental intensity maps of group 2 sulphide EH_3-22. a, Reflected light photomicrograph (same as Fig. 1d of main text), with corresponding colour-scaled (blue: low concentration; red: high concentration) elemental maps for S, Fe, O, Si and Ti (**b-f**, respectively), confirming that the interstitial phase is iron oxide. Small amounts of silicate melt may also get trapped, as shown in (**e**) and in previous Figure 3c. Note that the oxide phase is different from the host spinel phenocryst, as demonstrated by the absence of Ti in the interstitial oxide relative to the host (**f**), supporting our claim that the oxide phase exsolved from the immiscible sulphide liquid.

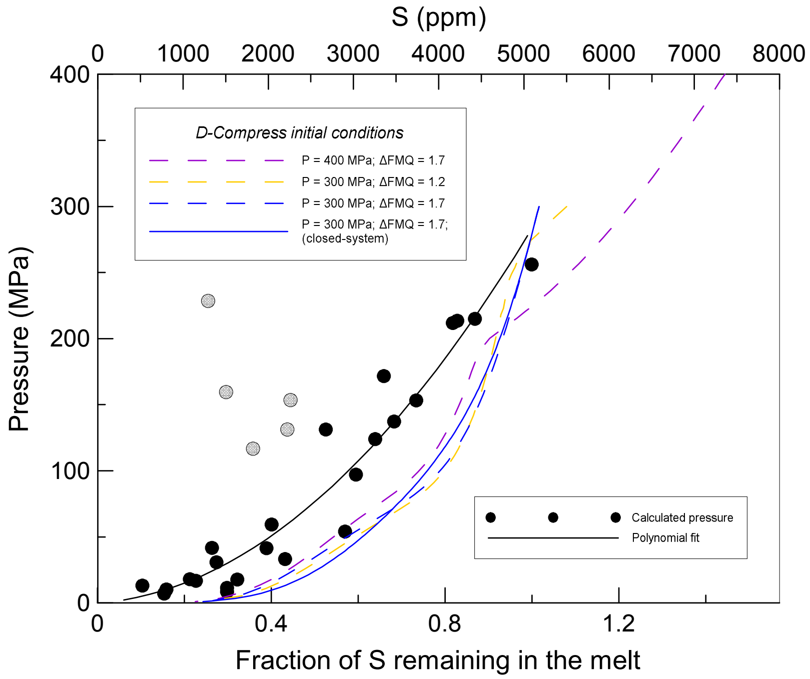

Supplementary Figure 5: Relationship between sulphur content and calculated vapour saturation pressures following Iacono-Marziano et al. (ref. [13]) (black circles). The black line shows the polynomial fit applied to the data (outliers shown by gray circles were excluded from this calculation). Dashed lines show various degassing scenarios computed with D-Compress (ref. [14]). The solid green line is a D-Compress output for closed-system degassing. Two of the D-Compress scenarios are also shown in Figure 2b of main text for their S and fO_2 outputs (300 MPa, open system).

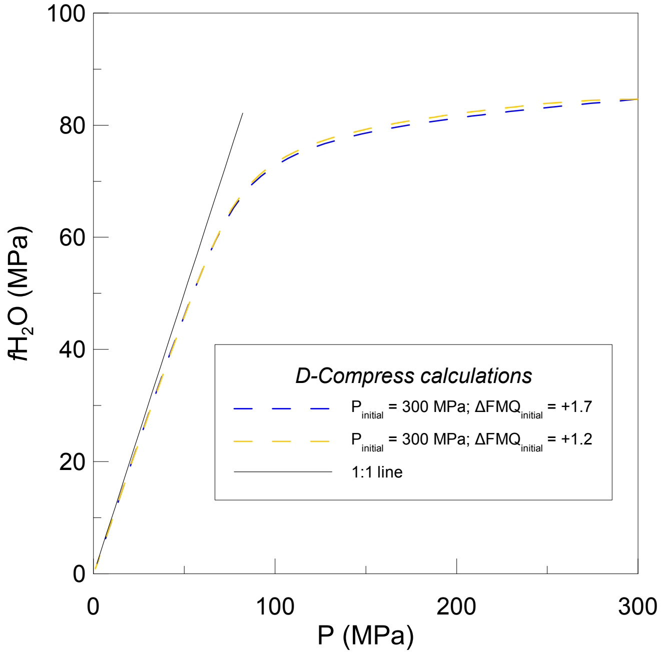

Supplementary Figure 6: Plot of water fugacity (fH_2O) against pressure (P) showing their convergence at low P. Therefore, using one or the other has virtually no effect in the isotopic fractionation models shown if Figs. 2a and 7 of the main text.

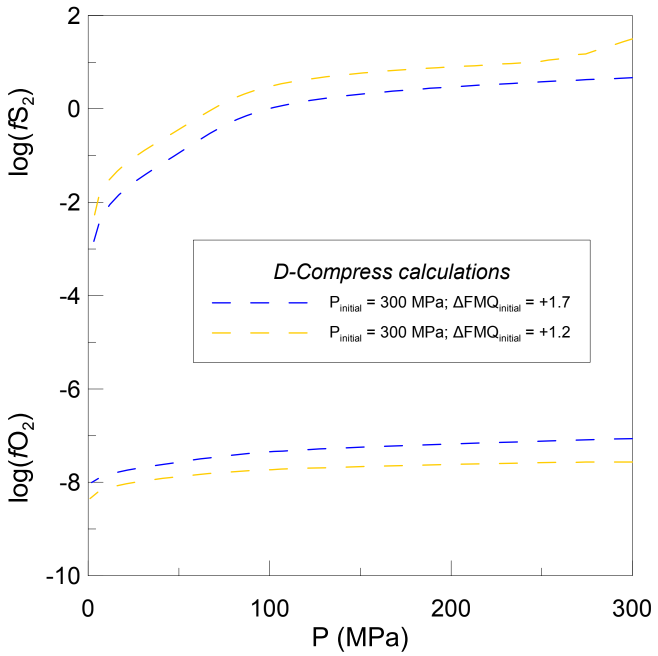

Supplementary Figure 7: *D-Compress* runs as in Supp. Figs. 2,3 showing the more pronounced decrease in fS_2 relative to fO_2 upon magma ascent and decompression, which may explain why some late-formed sulphides have a lower S contents and low totals, possibly due to the incorporation of O in the sulphide melt (see equation 2 in main text).

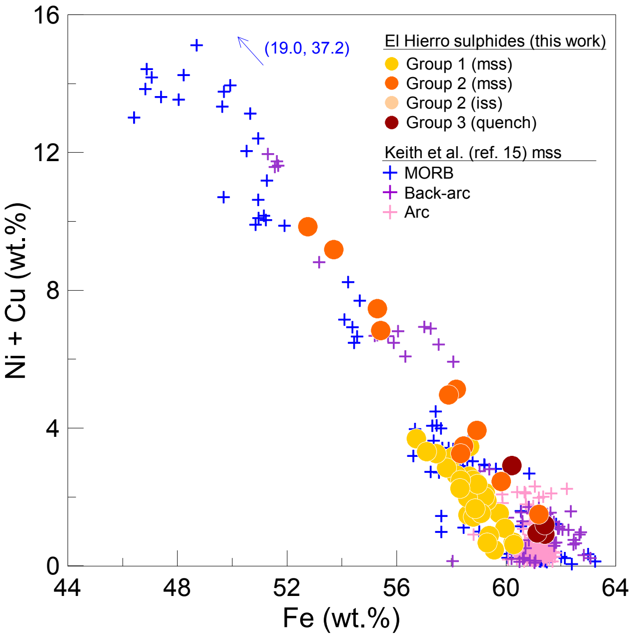

Supplementary Figure 8: Fe–Ni–Cu composition of El Hierro sulphides compared to mss from MORB (blue crosses), arc (pink crosses) and back-arc (purple crosses) settings[15], showing that they are more similar to arc and back-arc sulphides. The majority of MORB sulphides plot outside this figure, extending the trend continuously to 19.0 % Fe and 37.2 % Ni + Cu (mostly Ni; see Fig. 4 of main text). The axes are chosen as such because Ni and Cu are the major cations substituting for Fe (Fe+Ni+Cu represent >99.5% of cations in all samples). Note the two parallel trends exhibited by the group 1 sulphides (yellow circles) and that of groups 2 and 3 (orange and dark red circles, respectively) at slightly higher metal content, which may be explained by the lower overall S content of these sulphides (cf. Fig. 5a of main text and Supp. Table 2).

Standard	Phase	n	$\delta^{34}S_{measured}$ (‰)	± 1σ (‰)	$\delta^{34}S_{known}$ (‰)	$\Delta^{34}S_{IMF}$ (‰)
Balmat	py	6	19.6	0.3	15.1	+4.5
CDT	tro	3	-3.5	0.2	0	-3.5
CAR123	py	5	4.7	0.9	1.4	+3.3
Anderson	po	15	-1.0	0.4	1.4	-2.4

Supplementary Table 1: Instrumental mass fractionation ($\Delta^{34}S_{IMF}$) for the sulphide standards. Anderson pyrrhotite was analysed throughout the analytical session along with our samples, and we assumed the same IMF to correct the measured $\delta^{34}S$ values on our samples.

Supplementary References

1. Cabral, R. A. *et al.* Anomalous sulphur isotopes in plume lavas reveal deep mantle storage of Archaean crust. *Nature* **496,** 490–493 (2013).
2. Li, Y. & Liu, J. Calculation of sulfur isotope fractionation in sulfides. *Geochim. Cosmochim. Acta* **70,** 1789–1795 (2006).
3. Thomassot, E. *et al.* Metasomatic diamond growth: A multi-isotope study (13C, 15N, 33S, 34S) of sulphide inclusions and their host diamonds from Jwaneng (Botswana). *Earth Planet. Sci. Lett.* **282,** 79–90 (2009).
4. Fiege, A. *et al.* Sulfur isotope fractionation between fluid and andesitic melt: An experimental study. *Geochim. Cosmochim. Acta* **142,** 501–521 (2014).
5. Marini, L. *et al.* Effect of degassing on sulfur contents and δ34 S values in Somma-Vesuvius magmas. *Bull. Volcanol.* **60,** 187–194 (1998).
6. De Hoog, J. C. M., Taylor, B. E. & Van Bergen, M. J. Sulfur isotope systematics of basaltic lavas from Indonesia: Implications for the sulfur cycle in subduction zones. *Earth Planet. Sci. Lett.* **189,** 237–252 (2001).
7. Miyoshi, T., Sakai, H. & Chiba, H. Experimental study of sulfur isotope fractionation factors between sulfate and sulfide in high temperature melts. *Geochem. J.* **18,** 75–84 (1984).
8. Fiege, A. *et al.* Experimental investigation of the S and S-isotope distribution between H2O–S±Cl fluids and basaltic melts during decompression. *Chem. Geol.* **393–394,** 36–54 (2015).
9. Ohmoto, H. & Rye, R. O. Isotopes of sulfur and carbon. in *Geochemistry of hydrothermal ore deposits* (ed. Barnes, H. L.) 509–567 (Wiley, 1979).
10. O'Neill, H. S. C. Quartz-fayalite-iron and quartz-fayalite-magnetite equilibria and the free energy of formation of fayalite (Fe2SiO4) and magnetite (Fe3O4). *Am. Mineral.* **72,** 67–75 (1987).
11. Marini, L., Moretti, R. & Accornero, M. Sulfur isotopes in magmatic-hydrothermal systems, melts, and magmas. *Rev. Mineral. Geochemistry* **73,** 423–492 (2011).
12. Hulston, J. R. & Thode, H. G. Variations in the S33, S34, and S36 contents of meteorites and their relation to chemical and nuclear effects. *J. Geophys. Res.* **70,** 3475–3484 (1965).
13. Iacono-Marziano, G., Morizet, Y., Le Trong, E. & Gaillard, F. New experimental data and semi-empirical parameterization of H2O–CO2 solubility in mafic melts. *Geochim. Cosmochim. Acta* **97,** 1–23 (2012).
14. Burgisser, A., Alletti, M. & Scaillet, B. Simulating the behavior of volatiles belonging to the C–O–H–S system in silicate melts under magmatic conditions with the software D-Compress. *Comput. Geosci.* **79,** 1–14 (2015).
15. Keith, M., Haase, K. M., Klemd, R., Schwarz-Schampera, U. & Franke, H. Systematic variations in magmatic sulphide chemistry from mid-ocean ridges, back-arc basins and island arcs. *Chem. Geol.* **451,** 67–77 (2017).
