# Supplementary material for: Degassing-induced fractionation of multiple sulphur isotopes unveils post-Archaean recycled oceanic crust signal in hotspot lava
Source: Nat Commun. 2018 Nov 30;9:5093. doi: 10.1038/s41467-018-07527-w (PMC6269480; doi:10.1038/s41467-018-07527-w)
Supplement: Supplementary file 3 — Description of Additional Supplementary Files [file 41467_2018_7527_MOESM3_ESM.pdf]

Description of Additional Supplementary Files

File Name: Supplementary Data 1
Description: Sulphur isotopic data for melt inclusions and matrix glasses (Supplementary Table 1), sulphide inclusions (Supplementary Table 2), and characterization of our glass standard (Supplementary Table 3).

File Name: Supplementary Data 2
Description: Sulphur isotopic data for the sulphide inclusions measured by SIMS, as well as their chemical composition measured by EPMA.

File Name: Supplementary Data 3
Description: This spreadsheet contains the equations used to produce our sulphur isotope fractionation model, as well as annotations to indicate where the equations come from. The third tab contains the model outputs as shown in Fig. 2a.

File Name: Supplementary Data 4
Description: Characterization of our glass standard, P1326-2, by Isotope Ratio Mass Spectrometry (IRMS0.
